# Alcohol misuse within different socio-ecologies in rural communities of Botswana

**DOI:** 10.1371/journal.pone.0306542

**Published:** 2024-09-13

**Authors:** Refilwe P. Jeremiah, Masego Katisi, Odireleng M. Shehu

**Affiliations:** 1 Department of Health Promotions and Development, Graduate School of Human Interaction and Growth (GIHG), Faculty of Psychology, University of Bergen, Bergen, Norway; 2 Institute of Health and Participation, Faculty of Health and Social Sciences, Høgskulen på Vestlandet, Western Norway University of Applied Sciences, Bergen, Norway; 3 Department of Social Work, University of Botswana, Gaborone, Botswana; St John’s University, UNITED STATES OF AMERICA

## Abstract

Alcohol-related research in Botswana has rarely used a socio-ecological approach. This article presents a phenomenological in-depth analysis drawn from community mapping interviews (n = 23) collected among community leaders and service providers in one village in Botswana. The socio-ecological approach guided our research and analysis. This paper explored the influence of alcohol misuse within the cultural, familial, practices and legal frameworks in Botswana. Findings revealed patterns in alcohol misuse over time, the influence of alcohol misuse within different ecological systems, and their response to alcohol patterns as three global themes are discussed. The findings showed that alcohol misuse remains a major public health problem that trickles down from the community, and family systems to an individual, when there are with limited resources to address the alcohol misuse that exists. Recommendations to address alcohol misuse in Botswana include providing alcohol-free recreational places, more research on alcohol harm, and educating communities about alcohol harm.

## Introduction

Alcohol is a public health concern worldwide affecting individuals, families, and communities. Globally alcohol accounts for 3.3 million deaths yearly and for more than 200 diseases and injury conditions according to the Global Status Report on alcohol [1]. In Africa alcohol (mis)-use contributes to 6.4% of all deaths with a high prevalence of heavy drinking [1]. Alcohol misuse leads to several adverse influence that have short and long-term influence is on individuals’ health consequences such as injuries and diseases [1]. Alcohol is the leading risk factor for premature deaths and disabilities, contributing to non-communicable diseases and infectious diseases [2]. Alcohol can lead to risky behaviours in individuals due to environmental factors like availability, accessibility, and affordability of alcohol. [3]. In family systems, alcohol contributes to family disputes, and it trickles down to affect the community at large with negative behaviours such as drunken driving, sexual assaults, and other crimes [4]. Besides family, the immediate environment includes peers and neighbourhoods which can negatively influence individuals to engage in alcohol misuse [5]. For example, individuals may resort to alcohol consumption due to peer pressure for belonging. Cultural norms and beliefs in community systems significantly contribute to alcohol misuse [3, 6]. Community factors such as opportunities to purchase and consume alcohol and cultural values are found to influence an individual to use alcohol. Cultural values are a set of common societal norms and cultural practices in communities [7]. Therefore, this study explores the influence s of alcohol misuse within the different socio-ecologies in Botswana.

Most studies on alcohol misuse have focused on investigating individual patterns of alcohol misuse [8–10]. Few empirical studies have investigated alcohol misuse using broader perspectives like systems approach to go beyond just the individual, to conceptualise and analyse this challenge [5, 11, 12]. Most of these studies focus on prevention efforts and have been carried out in Western countries. There is a dearth of related or similar studies in Sub-Saharan Africa (SSA) [13, 14]. Given that alcohol misuse is almost an epidemic in many rural communities in SSA [15–17], there is a need for research that explores the characteristics and interaction of alcohol misuse within African local contexts and systems as there are major differences within the continent on the cultural and social context. In the present study, our focus is on Botswana.

Alcohol has been a significant part of Botswana culture for centuries and is associated with various cultural and social activities such as weddings and rituals. Traditional sorghum beer, known as *bojalwa ja Setswana*, was served among various ethnic groups during cultural, religious, and societal events, and was not sold [18, 19]. During such cultural activities, alcohol was drunk only by men, and it was prepared by women as it related to male power, authority, and patriarch [20]. Alcohol misuse in Botswana influenced the socio-economic development after the introduction of the cash economy during the colonisation era [18]. Over the years, the cash economy enabled Batswana’s financial independence and it influenced more people to drink alcohol in more quantities, spreading from adults to younger men and women [18, 19]. The cash economy also created a pathway for alcohol to be sold by women to make a living for their families. Many people in Botswana still drink beer, wine, spirits, and traditional brews [1]. Furthermore, studies in Botswana report that alcohol misuse is higher [21, 22] as compared to other African countries in the region. Alcohol consumption per capita of people who are 15 years and above is 8.4 litres of pure alcohol [1]. This paper defines heavier drinking as drinking more than 20 grams of pure alcohol per day and 40 grams of pure alcohol per day for me [23]. Whereas studies use alcoholic for heavier drinking for this paper alcohol misuse will be used. Botswana is known for its cultural heritage, and it prides itself on the traditions and beliefs of having chiefs (community leaders in rural villages). These chiefs implement and enforce the customary laws, including the use of alcohol during traditional ceremonies, together with police officers who implement and enforce common law [24].

The socio-ecological approach has commonly been used with the theory of ecological systems and social-ecological framework by Bronfenbrenner (1970s) [25]. However, this paper explores the influence s of alcohol misuse within the different socio-ecologies in Botswana. This approach is one of the many approaches within the group of complex systems theory [3]. The ecological systems framework was adapted to be the socio-ecological approach to show social and environmental dynamics when studying different topics related to human behaviour in social science for example by Michael Ungar on resilience [26, 27]. The socio-ecological approach is defined as “an interactional, environmental and culturally pluralistic perspective…which suggests a complexity in person-environmental interactions” within contexts [26]. The socio-ecological approach is about person-environment interactions, and it includes levels of (1) intrapersonal (individual). (2) interpersonal (family, peers), (3) organisational (community, schools), (4) environmental (cultural norms, physical environment) (5) and policy (laws and regulations). There has to be a goodness of fit among an individual and interactions between family, school, and community systems [26]. The socio-ecological approach is nested first within the interaction of the individual, social networks, and the environment. The available social and physical context in these systems can enhance an individual’s growth as they can influence a particular health outcome such as alcohol misuse [27].

Numerous studies contribute to alcohol and socio-ecological approach carried out in developed countries [4, 27–31]. Furthermore, there is a dearth of alcohol studies that used a socio-ecological approach in Sub-Saharan countries [32, 33]. Most studies generally use of the socio-ecological approach in other areas of research that are not alcohol-related. However, Lancaster and others conducted a quantitative study in Lilongwe Malawi on alcohol misuse among female sex workers to understand the social ecologies that influence hazardous drinking among female sex workers. The researchers found that half of the participants lived in an alcohol-serving location, also alcohol was associated with sex work duration. Participants used alcohol to reduce inhibitions and entice their clients during their sex work [32]. Although the socio-ecological approach is suitable for alcohol studies to our knowledge there are no similar studies in Botswana. Using a socio-ecological approach the article explores the influence of alcohol misuse within the different, socio-ecologies within communities.

## Materials and methods

The study uses data from an ongoing Doctoral study that explored the experiences of adult children of parents and caregivers with alcohol-related problems from one village in Botswana. The larger study aimed to understand how cultural and familial practices influence the perceptions and experiences of adult children of parents and caregivers with alcohol-related problems. This article is based on data from conducting community mapping with local stakeholders to understand the context of alcohol misuse in Botswana’s largest rural district. The study was conducted in a village with a history of alcohol misuse [18] allowing for a more comprehensive understanding of the issue as there is limited research done. Data were collected carried out from February 2022 to March 2022. The study mapped the socio-cultural community context of alcohol misuse in the village. Methods used to gather data included focused group discussions with four Village Development Committee (VDC) members; in-depth interviews with fifteen key informants including two police officers, a junior school guidance and counselling teacher, a primary school head teacher, two social workers, four village chiefs, five traditional alcohol sellers (see Table 1). Nineteen participants took part in this study, both males and females aged twenty-five years to thirty-three years for young adults and elders in the community aged forty years to sixty-five years.

**Table 1:** Details of participants and data collection methods.

Role	Method	MALE	FEMALE
Social workers	In-depth interviews		2
Police officers	In-depth interviews	2	
Primary school headteacher	In-depth interview		1
Junior school guidance teacher	In-depth interview	1	
Senior chief	In-depth interview	1	
VDC members	Focus group discussion		4
Traditional alcohol sellers	In-depth interview		5
Sub-chiefs	Focus group discussion		3
Pastors	In-depth interview	4	
Total		23	

The social workers and VDC members in the village acted as gatekeepers in recruiting participants and snowball sampling was also employed. Participants were purposively selected to participate in this study, given that they know the community very well or interact a lot with alcohol issues. Due to the uniqueness of the participants, the first author who was collecting data was given access to information that could identify participants during and after data collection. Participant observation was employed to conduct community mapping in which knowledge is co-produced with local participants who know the values of indigenous knowledge. The first author had extended field time by walking around the village and observing alcohol drinking habits in shebeens and interacting with alcohol buyers at these shebeens as bar outlets were closed during covid-19 lockdown. Shebeens are unlicensed homes that sell traditional brews in the community.

Participants read the written informed consent of the study. The first author described the purpose of the study, participants were informed about foreseeable risks and benefits of participants and that they should not feel any pressure to talk if they felt the study was putting them at risk. Participation in the study was voluntary, and participants were informed that they had the right to withdraw from the study at any time. Data from participants were handled with confidentiality and all characteristics from the audio recorded were replaced with pseudo codes following the University of Bergen General Data Protection Regulation and Personal Data Act. Participants were informed about consenting that their information be transferred from Botswana to Norway as part of research collaboration and publication for the Ph.D. study of the first author. The authors would store data on the University of Bergen SAFE server. When participants verbally agreed to the information, they signed informed consent forms before participating in the study.

### Data analysis

Data were audio recorded in Setswana (local language), with permission from participants. Data were then transcribed and translated into English. The first author did member checking where some participants were asked to verify the accuracy and completeness of data collected by reading their own transcribed scripts. Nvivo 12 scientific software was used to manage data. Data were inductively coded as the inductive approach provides a broader and more expansive analysis of the entire data [34]. Thematic Network Analysis was used to analyse data using the steps suggested by Attride-Stirling [35]. Similar or related codes were grouped into basic themes clustered into organising themes and global themes. The findings are therefore presented using global and organising themes (see Table 2). The global themes are (1) patterns in alcohol misuse over time (2) the influence of alcohol misuse within different systems (3) system responses to alcohol patterns. When analysing data, it was evident that there are overlaps in the individual, family, and community systems.

**Table 2:** Findings analysis table.

Codes	Basic themes	Organising themes	Global themes
Types of traditional brews Ways of making alcohol sorghum beer	Changes in brewing alcohol	Alcohol misuse within Botswana’s cultural systems	Patterns in alcohol misuse over time
Alcohol sorghum beer used in traditional ceremonies	Traditional uses		
Alcohol sorghum beer drunk by men only Historic influence of traditional alcohol	Users & influence		
Alcohol sales used to pay for school fees to make a living Harmful ingredients with alcohol sorghum beer	Alcohol as a source of income	Trend of alcohol misuse in the cash economy	
Drinking alcohol as a daily normality by different age groups Behaviour of alcoholic customers	Current alcohol drinking habits		
Destitute persons trading social welfare benefits for alcohol	Abuse of social welfare services for alcohol		
Child neglect Intergenerational alcohol misuse Alcohol leading to misunderstandings Children imitate their parents. Child truancy due to lack of parental care	Parents not taking responsibilities	Alcohol misuse in family system	Influence of alcohol misuse within different systems
Children born in alcoholic homes becomes like their parents Other children born in alcoholic homes are not using alcohol	Children learn by imitating parents		
Parents’ alcohol drinking led to child’s absenteeism Selling alcohol in homes lead to poor school performance of children	Parental alcohol misuse affects students’ education	Alcohol misuse within the education system	
Alcohol misuse by students in boarding schools Alcohol leading to school dropouts Students threaten teachers at bar outlets	Alcohol use among students		
Schools and teachers enforce discipline on students	School/teacher response to students alcohol misuse		
Alcohol causing fights and threats Alcohol leading to theft Alcohol misuse leading to rape Alcohol leading to defilement Alcohol causing deaths	Alcohol and criminal activities	Interplay of individual, family and community dynamics	
Alcohol leading to unplanned pregnancy Alcohol leading to teenage pregnancy	Alcohol leading to high-risk sexual behaviours		
The law protects children from buying or selling alcohol no laws regarding students drinking outside school	Children’s Act	Traditional and legal system	Systems responses to alcohol patterns
Alcohol policy not addressing the actual drug issues	Alcohol Levies		
Traditional alcohol is regulated by chiefs	Customary law on alcohol misuse controls		
Social workers providing alcohol educational campaigns Role of the church in alcohol control in the community	Role of community stakeholders	Stakeholders’ involvement in alcohol related campaigns	
Back to school programme for school dropouts PACT program that addresses student issues	School based activities		
Music competitions used as alcohol educational activities Collaboration of stakeholders needed in alcohol related campaigns	Community organised activities as a resource		
Bar outlets as forms of entertainment Villagers do not welcome outsiders to help change behaviour	Resource challenges		

### Ethics

Ethical clearance was obtained both in Norway and Botswana. In Norway ethical clearance was acquired from the University of Bergen Data Protection Ombudsman and the regional ethics committee (*Regionale komiteer for medisinsk og helsefaglig forskningsetikk*, REK). In Botswana, ethical clearance was obtained from Ministry of Health and Wellness. At the organisational level, extra verbal authorisation was obtained from community gatekeepers while written permits were obtained from the Botswana Police Department, and the Social and Community Development (S&CD) agency. Part of the ethical procedure included respecting and following cultural protocols, for example, when interviewing the village sub-chiefs, the first author had to wait for a day when she was not wearing trousers. It is against Botswana culture for a woman to wear trousers to enter the kgotla (customary traditional court). Researchers followed the regulations of the General Data Protection Regulation (GDPR) at the University of Bergen. We securely stored all personal data in the University of Bergen SAFE server. At the end of the Ph.D. project term, all data will be anonymised and stored in the University of Bergen Open Research space.

### Trustworthiness, quality assurance, and verification

The study’s credibility was ensured through data collection, management, analysis, and reporting, following Lincon & Guba’s criteria for trustworthiness, focusing on credibility, dependability, and confirmability, in line with qualitative studies interviews. The study’s first author used reflexivity and bracketing to prevent bias by ensuring her prior knowledge of participants did not influence data collection and results. This allowed co-authors’ extensive experience in Social Work and Psychology research to set aside pre-existing knowledge and personal views. To enhance transferability, researchers enrolled diverse participants with characteristics like the larger population, such as educational background, sex, and age. Further, the findings of the study can be used to inform research done on similar villages and other Sub-Saharan African countries like Botswana. Participants were encouraged to be honest in their responses to the study about a phenomenon of interest. The first author, a clinical social worker with experience in qualitative data collection conducted interviews. Additionally, the researcher conducted extended field time to understand the livelihood dynamics of alcohol-misusing people by visiting traditional brewing homes. To fill the gap between participants’ interviews and the researcher’s knowledge and thinking, journaling was done to collect observed field notes. The field notes were incorporated into data analysis to produce rich contexts that represented participants. Transferability was ensured by interviewing a representative of all stakeholders in the village to understand the rich contextual experiences of alcohol misuse in the village. Data analysis included the other two researchers, who are social work professors. Possible biases were discussed to ensure adequate resolution. The first author read the data set multiple times to understand participants’ experiences.

The first and second authors read raw data separately and discussed possible themes. Following this, the first author separately coded the data and discussed it with the second author. The other authors continuously gave feedback on the codebook. They compared generated themes to find the most accurate descriptions of participants’ experiences. Disagreements were resolved through discussions, and findings were supported with quotes from participants. The process ensured a comprehensive understanding of the participants’ experiences. The authors used Morse et al. [36] steps to ensure the quality and verification of phenomenological research data and findings. Methodological coherence, appropriate sampling, simultaneous collection, analysis, theoretical thinking, and developing theory were followed to ensure the authenticity and verification of the study’s findings.

## Findings

### Patterns in alcohol misuse in Botswana over time

This section explores the global patterns in alcohol misuse in Botswana, focusing on two organizing themes: alcohol misuse within Botswana’s cultural systems and the trend of alcohol misuse in the cash economy, highlighting the influence of these changes on individual, family, and community systems.

#### Alcohol use within Botswana’s cultural systems

This section explores the history of brewing alcohol in Botswana, focusing on its traditional uses and influence. Alcohol misuse has been a part of Botswana’s tradition, with various types introduced over time. These include traditional *sorghum beer*, *khadi*, *stopoti*, *morula*, *power*, *skhothan*i. and c*hibuku* as a commercialised traditional beer. In this study, we label the original traditional home brew as *bojalwa ja Setswana*. Participants noted that the current traditional sorghum beer differs from the past due to higher alcohol content. They shared original methods of making traditional sorghum beer. Seller 3 described ways of making alcoholic sorghum beer:

*Traditional sorghum beer, in its essence, is a sorghum meal fermented. We use traditional pots or bigger containers. The sorghum seeds are soaked in water until they show little sprouts. After that, they are taken for grinding to make them for brewing. Add boiled water and let it ferment. Wait for three days and add sorghum mixture as a way of preparing the traditional sorghum beer. We only use sorghum meal, nothing harmful is added to it.*

Historically, traditional sorghum beer was used in traditional ceremonies such as weddings, initiations, and the inauguration of chiefs just to mention a few. VDC member 4 added: *During celebrations like weddings*, *you would never miss big containers of traditional sorghum beer*. *Traditional sorghum beer was always there*. *Indeed*, *alcohol misuse was deemed very important in society*. The alcohol sorghum beer was also used by traditional doctors for rituals and healing. Since it was made from sorghum and was assumed not harmful it could be given to children when they were sick, Pastor 4 explained:

*When children developed illnesses like measles, parents would give them traditional sorghum beer to drink. If it was chicken pox traditional sorghum beer would be rubbed on the child’s body and given to drink.*

Alcohol sorghum beer was drunk by men only and prepared by women during cooperative communal help in the community as a recognition for their hard work in the fields. Most participants indicated that alcohol sorghum beer had mild alcoholic influence on individuals. Chief 1 remarked, comparing it to other types of brews:

*We would spend the whole day drinking alcoholic sorghum beer without assaulting anyone. As for modern alcohol, I really don’t understand how people end up being involved in fights and causing harm to one another.*

#### Patterns of alcohol misuse in the cash economy system

Participants were asked to comment on patterns of alcohol misuse in the cash economy. Most of the responses pointed out that there are people that sell alcohol for generation of income and that others drink it for self-fulfilment. Under this theme topics that came out from the interviews included alcohol as a source of income, current alcohol drinking habits, and the abuse of social welfare services by alcoholic beneficiaries.

While communities used to share alcohol without any monetary exchange, data reflect that this seemed to have changed over time. Some brewers use alcohol as a source of income, particularly women. Several participants stated that some members of the community sell alcohol to pay school fees and earn a living. VDC member 4 added: *Women sell alcohol to raise money to buy school uniforms*, *and shoes and to pay school fees for their children*. To meet the market demand brewers started to use unnatural ingredients to activate it to brew faster and to increase the level of alcohol content. Police officer 1 shared: *Sellers use different things like cell battery powers*, *and certain spirit drinks to speed up the reaction when making home brews*. It appears that the introduction of the cash economy has influenced present alcohol consumption patterns. Participants said they observe that there is increased access to alcohol to different age groups as compared to the earlier cultural practice where only men were allowed to drink on special occasions. It seems it is now normal for women to drink. This was illustrated by several participants. Pastor 1: *People of different ages and gender easily drink these days unlike in the past since alcohol is sold everywhere*. Pastor 4 added: *It is now so much embedded in the culture and current values and has become a way of life*. Participants said that alcohol drinkers’ behaviour changes dramatically after drinking alcohol due to the high alcoholic content in recent brews. A social worker shared that some destitute persons misuse government welfare packages such as food baskets in exchange for alcohol. Social worker 2 attested:

*We would enrol a destitute old woman with 12 dependants. Due to the number of family members, instead of depositing P500 (around $42) into the coupon, we would deposit P1000 (around $83). Instead of buying food, some family members would use the money on alcohol. In other families when they receive their food packages today the next day there is nothing on the table. They sell them so that they can afford to buy alcohol.*

### Influence of alcohol misuse within different systems

It is observed that alcohol has affected different systems within the community. Alcohol practices within the family system, alcohol misuse within the education system, and alcohol misuse in the larger community system are organising themes discussed in this section.

#### Alcohol practices within the family system

Alcohol has influence on the family system, under this theme topics that came out from the interviews included parents not taking responsibility, and children learning by imitating parents. It is observed that parents who use a lot of alcohol are lax in carrying out their parenting responsibilities. Further, most participants emphasised that some parents in the community neglect their children and leave them with grandparents to raise. The primary school head teacher said: *In other cases*, *we have realised this fact*, *truant children*, *in most cases it is because they lack monitoring back home and there is no parental care because they stay with their grandparents who are old and not able to provide care well*. Further, parents’ negligence in carrying out their parental responsibilities has resulted in intergenerational alcohol misuse within families as illustrated by Pastor 4: *It is very normal looking at the setup*. *You will find that the mother is there drinking and the father*, *the daughter is drinking*, *the boyfriend of the daughter also is drinking*, *it is normal*.

It is also alleged that alcohol misuse leads to misunderstandings in families as attested by the Main chief: High level of *alcohol misuse affects families*. *In the case where both parents overdrink alcohol family misunderstandings occur often*. Moreover, one of the influences on the family system is that children imitate their parents when they grow up in families that sell or misuse alcohol. They too become alcohol misusers as alluded by these participants: VDC member 3 shared: *Children growing up in families that abuse alcohol does not attend their school days as expected*, *and they end up drinking alcohol*. Pastor 1 also added:

*I have a cousin who was very good with traditional music, but now he is just home and useless because of alcohol addiction. He grew up in a home where his mother was selling alcoholic sorghum beer. In that family, all members drink; be it those in primary, junior, and secondary school, the old people, and those that are suffering from blindness. Even children are given alcohol to drink.*

On the contrary, some children are exposed to the use and sale of alcohol within their homes, and yet they do not copy their parents and do not use alcohol. VDC member 1 explained: *Children born into some families where alcohol is sold do not necessarily end up drinking*. *When we grew up*, *my mother used to brew and sell alcohol*, *but me and my siblings do not drink alcohol*.

#### Alcohol misuse within the education system

Alcohol seems to affect children’s education. Under this theme topics that came out from the interviews included how parental alcohol misuse affects students’ education, alcohol use among students, and school or teachers’ responses to students’ alcohol misuse. Parents alcohol misuse is found to affect children who are students. The extent of alcohol drinking by some parents led to children’s absenteeism and affected children’s education. The primary headteacher explained:

*Some of the absenteeism is alcohol-related; a parent leaves the home in the morning without making sure that the children are well prepared for school, like I said we visited one homestead, and the children had no idea where their mother could be.*

Further, participants stated that alcohol sales in the homesteads done by parents influence students’ poor performance at school. Village Development Committee (VDC) member 2 explained: *Sometimes children do to go to school*, *instead parents will give the child errands to sell alcohol*, *or maybe go and buy alcohol brewing ingredients*.

It is observed that alcohol misuse affects the education system in terms of teaching and learning outcomes, as well as students’ retention. Some students use alcohol within boarding schools, which is against school regulations as attested by the focus group with VDC members. The guidance and counselling teacher added they have alcohol misuse as one of the main problems in their boarding school. Some participants explained that some of the school dropout cases were related to the extent of alcohol misuse in families. The Guidance and counselling teacher: *As teachers we have observed that school dropouts tend to abuse alcohol as compared to other students*. *A significant number of them end up not completing their studies and go all out there to spend time in bars*. Additionally, alcohol misuse contributes to some students having bad behaviour towards teachers. The Guidance and counselling teacher further explained an incident where students threatened one of the teachers at a bar outlet:

*I remember one case student who harassed one of the teachers at the drinking spots saying he always reports things that happen outside school back to school authorities. They grabbed him by his clothes and tried to fight him as a way of revenge since he once punished one of them.*

On the Contrary, guidance and counselling teacher said the school system enforces discipline on students who are found using alcohol or are seen at bar outlets. He attested: *We had cases where a group of boys was found at bars and drinking spots*. *The next working day they received their punishment for such behaviour*.

#### The interplay of individual, family and community dynamics

Alcohol misuse also has a significant influence on the larger community. The topics in this organising theme include alcohol and criminal activities, and alcohol leading to high-risk sexual behaviours in the community. There are alcohol-related crimes reported to the police that include incidents of fights and threats: Police officer 2 said: *There are people who usually misbehave after drinking alcohol in bar outlets*. *Some of them end up fighting*, *and some use dangerous weapons like knives*. Other participants reported cases in which alcohol misuse leads to stealing. VDC member 3 shared: *There are lots of common theft issues where excessive alcohol users are involved*. According to participants, alcohol usage is also linked to rape and defilement in society. Social worker 2 said: *One of the challenges is defilement and rape*. *Currently*, *we have more than 25 cases of defilement in this area which are alcohol related*. However, some parents do not report these cases at the police department against those responsible for defilement. As a result, these perpetrators are not punished according to the law which left children still vulnerable and not protected. Pastor 4 stated:

*We observe cases of sexual abuse in the community. We have children starting from primary up to standard 5 engaging (7 to 11 years old) in sexual activities with very older men and you can find standard 7 (13 years old) pupils dropping out of school. Parents do not take legal action on such matters.*

Similarly, other participants reported that there were criminal activities associated with alcohol, even leading to deaths in the community. Pastor 4 shared: *There was a case of murder that was reported in this area*, *a young man was killed at bar outlets*. While VDC member 3 emphasised:

*A man was killed on the spot at bar outlets for buying another man´s girlfriend alcohol. When the boyfriend realised that his girlfriend accepted alcohol from that man, he got angry to the point where he used something sharp to kill the intruding man.*

It was also observed that alcohol misuse leads to high sexual risk behaviours resulting in unplanned pregnancies according to participants. Pastor 3 said: *Unplanned pregnancies are also part of the risks associated with alcohol misuse*. The guidance and counselling teacher gave an example of a teenage girl who got pregnant due to the influence of alcohol: *I remember one case of a student*, *who dropped out of school without any stated reasons*, *and she spent more time at bars*, *after a year she got pregnant and never returned to school*.

### Systems’ responses to alcohol patterns

Participants highlight that at the community level, all stakeholders need to act to address the influence of alcohol misuse in the community. There are already a few strategies put in place at educational system, society at large, and legal systems to regulate alcohol misuse in the community. This section discusses traditional and legal systems, and stakeholders’ involvement in alcohol misuse campaigns as their contribution towards the influence of alcohol misuse in the community.

#### Traditional and legal system

In Botswana, there are traditional and legal systems in place that include the Children’s act, alcohol levies, and customary laws of alcohol control. Under this organising theme topics that came out from the interviews included the Children’s Act, alcohol levies, and customary law on misuse controls. Participants reported that the government has policies and regulations set through civil and traditional systems. social worker 1 said: *According to the law in Botswana*, *we have Children’s Act*, *which criminalises parents from sending their children to buy or sell alcohol and other substances*. However, the guidance and counselling teachers said that there are no specific education laws that regulate children’s drinking outside school premises. It is only within the school premises that this is clearly stated. He said:

*We don’t have clear and binding laws to monitor them outside school. We sometimes try, since last year and the year before, we had cases where a group of boys were found at bar outlets. We could identify them. When they came to school, we sat with them and talked. Even though we do not have any laws that govern us to act concerning behaviours outside school, it still affects us.*

Participants explained that the increase in alcohol prices that were introduced through Botswana alcohol levies to manage alcohol misuse did not produce the desired outcomes. This is illustrated by Police officer 2: *The alcohol levy was introduced by the former president*, *with a hike in alcohol prices*, *closing unregistered depots*, *etc*. *However*, *the increase did not yield behaviour change or a reduction in alcohol intake*.

At the community level, it is the responsibility of village chiefs to regulate the production and sale of traditional homemade brews. The chiefs are guided by the Traditional regulation act Police officer 2: *Traditional beer is regulated by chiefs they are the ones to issue brewing permits*.

#### Stakeholders’ involvement in alcohol misuse campaigns

The role of community stakeholders, school-based resources, community organised activities are identified as key resources and resource challenges are sub-themes covered under this organising theme. Data suggest that community stakeholders play an important role in the enforcement of the traditional legal system. Social workers shared some alcohol campaign activities: *In the previous year*, *we had an educational campaign tour with the district commissioner’s department*. *As a result*, *we noted some improvements in the youths’ behaviour*. There are school-based activities that contribute to the responses to alcohol misuse in the community, such as back-to-school programmes for school dropouts as attested by Guidance and counselling teacher: *What happens is after a student has dropped out of school at a senior level looking at their specified age range*, *we readmit any child who wants to come back to school again*. It was described that schools also have Peer Approach and Counselling by Teens (PACT) programme that addresses students’ psychosocial challenges and teaches them social life skills.

Pastors also explained their role in the community. They claimed that providing prayer, deliverance, and counselling to community members helps ease alcohol abuse in churchgoers. Similarly, participants said they used to be community-organised activities that helped address alcohol misuse in the community. For example, alcohol-free campaigns such as music competitions and football tournaments encouraged community members to have alcohol-free entertainment, but these do not exist anymore.

For stakeholders to address alcohol use and misuse in the community, participants said there is a need for all stakeholders to collaborate and work together on alcohol-related projects. Pastor 1 attested: *Alcohol reduction in this village can be achieved through the collaboration of all community stakeholders*. Although there are resources that stakeholders use to address alcohol misuse in the community, there are challenges such as a lack of recreational places and bar outlets used as a form of entertainment as explained by Police officer 1: *There are no entertainment parks except indulging in alcohol at bars*. Similarly, another identified challenge is that villagers do not welcome outside players to help them change their behaviour toward alcohol misuse. Pastor 4 stated:

*Normally, you cannot come from outside and easily penetrate and bring people out of this pattern. Because alcohol is something that somehow has become part of them*.

## Discussions

The current study used the socio-ecological approach to explore the influence of alcohol misuse within the different systems in Botswana. The findings indicate that there is hazardous alcohol misuse in the village which trickles down from the community, through the family down to the individual in alignment with what Ungar said about how individuals’ behaviour is influenced by their broader environment [26]. This will be reiterated throughout the discussion to demonstrate how the study compares to similar studies’ findings to evaluate their significance. The findings reveal how systems such as family, education, and community systems interact with each other and influence an individual alcohol misuse. this is observed in how family norms and values get influenced by cultural norms and have an influence on an individual’s behaviour. Further, an individual’s behaviour contributes to the family setting, and influences cultural norms and educational outcomes.

There is an intergenerational drinking culture where children drink with their parents and caregivers. This courses a conflicting interplay between school, young people, and family. Students play truancy and miss school because of drunkenness, at the same time, they are taken advantage of by the community members [37], raping and exploiting them. Parents responsible for these at-risk youth ignore their education and avoid meeting teachers to help build young people’s morality and character. The irony is that these young people become dangerous to adults from families in the community and attempt to kill them. This is what Ungar [26] calls systems According to previous studies [5, 8] there are contextual risk factors for alcohol misuse among youth presented by their family and peers, thus, youth learn these behavioural patterns from their systems. Sebeelo [21], who asserts that extended family members and caregivers share traditional brews with children in the same compound, supports this assertion. However, because the study was focused on community mapping and participants were community stakeholders, the findings (adults) make no mention of youth experiences with alcohol misuse as influenced by family and peers. Therefore, further research is needed to provide insight into this area and document the influence of such systems on young people.

The findings showed a complex interaction between individual children and family. Parents neglect their children and leave them with grandparents who quite often are under the influence of alcohol. This could be attributed to limited jobs in the study area/setting due to the geographic setting. As a result, parents, particularly mothers, frequently depart to seek employment in towns and cities. Consequently, the people who remain in the villages spend most of their time in home brewing establishments and have little time to spend with children. Thus, children end up not having a relationship with their parents. Similarly, research attests that having a good communication relationship between parent-child can buffer a child from being involved in risky behaviours such as alcohol misuse [38] Thus, the findings provide a deeper understanding of the importance of parent-child communication where children are encouraged to be honest about their educational progress and personal issues. This will decrease the risky behaviours children engage in, such as attacking their teachers at bar outlets.

There are strong social norms surrounding alcohol consumption as part of social activity in the community which has a strong influence on individuals to end up using alcohol. Alcohol is deemed part of Tswana culture as it is used for leisure and seen as a way of life. This is in line with [21] study that also found that alcohol is a way of life in Botswana and Batswana liked to drink alcohol as it was a social activity that brought friends together. Although there is a trend of alcohol consumption among women in the modern times/contemporary world, culturally this has not been a fully accepted phenomenon in Botswana. In corroboration, a study on alcohol abstinence and drinking among 20 African women from different parts of African countries found that although there was moderate alcohol consumption among women, the African culture does not accept women drinking alcohol [39]. Even so, the observations made around the home brews demonstrate that there were older women who were not working but drinking traditional homemade brews. The study findings point to the necessity for further research to explore women drinking and what their experiences are like in this village. Further research is needed to explore the issue of women and alcohol misuse in Botswana using a socio-ecological approach.

The findings indicate that the availability of many alcohol outlets in the community is found to increase the drinking culture which is found to be a source of socialisation in the community. According to the observation done by the leading researcher, there were ten bar outlets and about fifteen traditional home brewing places by the time data was collected in a population size of 8,342 people. This influences the reported highly hazardous drinking observed in the community due to the readily available alcohol. Although the alcohol outlets are used as a source of socialisation, the findings reported criminal activities such are alcohol leading to death, rape, and assaults that are perpetuated at these places. According to [40] where there are more alcohol outlets there are more social problems such as violent assaults. This calls for policymakers and practitioners to investigate how alcohol influences the criminal activities that exist in the community as interventions to address alcohol misuse are addressed.

While the study’s findings revealed certain dangerous patterns of alcohol misuse, there is also information that suggests some ways to address the influence of alcohol misuse in the community. The findings show that traditional and legal systems, as well as stakeholder involvement in alcohol abuse campaigns, all contribute to curbing the influence of alcohol misuse in the community. Programmes that target neighbourhood planning, zoning, and licensing are among the most effective approaches to reducing socioeconomic gaps in alcohol-related outcomes [41] as they address alcohol misuse in community system which will trickle down to the family system and the individual system. Overall, the findings highlight the importance of stakeholder collaboration in alcohol-related efforts.

## Strengths and limitations

This study has made a valuable contribution to the under-researched field of alcohol misuse in rural regions. Furthermore, the study findings provide valuable insights for researchers, practitioners, and policymakers, enabling them to develop evidence-based treatments for addressing alcohol-related issues in rural areas. These interventions can also be implemented in other regions of Botswana and Sub-Saharan countries. Additionally, the study used a socio-ecological method to explore how alcohol consumption affects individuals, families, and communities. This community mapping has created an understanding of the community context for the subsequent stage of the Ph.D. project. The next phase of the Ph.D. project will be to explore the experiences of adult children of parents and caregivers with alcohol-related problems in the same village. Nevertheless, the study encountered some limitations. Data collection was conducted during the COVID-19 pandemic. Retail stores selling alcohol were temporarily shut down to comply with COVID-19 regulations. Consequently, the business proprietors were not interviewed. Furthermore, recruiting participants using the snowballing method may have a potential for biases as participants with the same characteristics may be recruited.

## Conclusion

The study explores the impact of alcohol misuse on public health in Botswana, focusing on the socio-ecological approach. It highlights the intergenerational drinking patterns in the village, influenced by historical alcohol use, and the negative influence on families, leading to low educational performance and a lack of parental-child relationships. The high availability of alcohol outlets in the community also contributes to social problems such as risky behaviour and crime just to name a few. The study suggests creating alcohol-free recreational facilities in villages to address social issues influenced by excessive alcohol use. The study adds to the body of knowledge that aims to guide future research and practice in developing influence systems to address alcohol misuse in the country.

## Supporting information

S1 Checklist Inclusivity in global research. (DOCX)

S1 File Annex 5 (Interview guide for stakeholders). Interview Guide For Participating In The Study.
(DOCX)
