# Supplementary material for: Alcohol misuse within different socio-ecologies in rural communities of Botswana
Source: PLoS One. 2024 Sep 13;19(9):e0306542. doi: 10.1371/journal.pone.0306542 (PMC11398658; doi:10.1371/journal.pone.0306542)
Supplement: S1 Checklist — (DOCX) [file pone.0306542.s001.docx]

Inclusivity in global research

PLOS’ policy on inclusivity in global research aims to improve transparency in the reporting of research performed outside of researchers’ own country or community and ensures that PLOS publications reporting global research adhere to high standards for research ethics and authorship. Authors of relevant research articles may be asked to complete the questionnaire below, which outlines ethical, cultural, and scientific considerations specific to inclusivity in global research. This questionnaire may be requested when researchers have travelled to a different country to conduct research, if research uses samples collected in another country, research with Indigenous populations or their lands, or if research is on cultural artefacts. Researchers travelling to another country solely to use laboratory equipment will not normally be required to complete the questionnaire. However, the questionnaire can be requested at the journal’s discretion for any submission – if you have been requested to complete this questionnaire by the PLOS journal you submitted to, please do so.

Please complete the questionnaire below and include this as a Supporting Information file with your manuscript. Note that if your paper is accepted for publication, this checklist will be published with your article in the supporting information files. Please ensure that you reference the checklist in the main body of your manuscript. We suggest adding a subsection ‘Inclusivity in global research’ to your Methods section and adding the following sentence: “Additional information regarding the ethical, cultural, and scientific considerations specific to inclusivity in global research is included in the Supporting Information (SX Checklist)”

The questions have been designed to be applicable to a wide range of study types, and there are subsections for both human subjects research and non-human subjects research. If any of the questions are not relevant to your research please mark them as “N/A” as appropriate.

**Ethical considerations, permits and authorship**

*This section is applicable to all research types.*

Provide details as to who granted permissions and/or consent for the study to take place in the Methods section of your manuscript. This should include the names of **all** ethics boards, governmental organizations, community leaders or other bodies that provided approval for the study. If individuals provided approval refer to these people by their role or title but do not list their name(s).

Reported on page number: page 10 of the manuscript describes ethical approval for the study in Norway was from the regional ethics committee (*Regionale komiteer for medisinsk og helsefaglig forskningsetikk*, REK). In Botswana, ethical clearance was obtained from Ministry of Health and Wellness. Ethical approval letters are attached as supplementary materials

If there were any deviations from the study protocol after approval was obtained please provide details of these changes in the Methods section of your manuscript.
Did this study involve local collaborators that are residents of the country where the research was conducted or members of the community studied? If you do not have any authors from said communities, please provide an explanation for this below.

The study was conducted in Botswana where the first, 2^nd^ and 4^th^ authors are citizens. The first author who is a Motswana collected data using the local language Setswana. She is not from the community where data were collected. However, she worked with the community’s social worker, a gatekeeper.

Reported on page number: There was none

Everyone listed as an author should meet PLOS’ criteria for authorship and all individuals who meet these criteria should be included in the author byline, rather than the acknowledgments. For further information please see the journal’s Authorship Policy.

**Human subjects research (e.g. health research, medical research, cross-cultural psychology)**

Did you obtain written informed consent from a representative of the local community or region before the research took place? How did you establish who speaks for the community? Details of written informed consent obtained from study participants should be reported separately in the Methods section of your manuscript.

Page 7: Participants read the written informed consent of the study. The first author described the purpose of the study, participants were informed about foreseeable risks and benefits of participants and that they should not feel any pressure to talk if they felt the study was putting them at risk. Participation in the study was voluntary, and participants were informed that they had the right to withdraw from the study at any time. Data from participants were handled with confidentiality and all characteristics from the audio recorded were replaced with pseudo codes following the University of Bergen General Data Protection Regulation and Personal Data Act. Participants were informed about consenting that their information be transferred from Botswana to Norway as part of research collaboration and publication for the Ph.D. study of the first author. The authors would store data on the University of Bergen SAFE server. When participants verbally agreed to the information, they signed informed consent forms before participating in the study.

How did members of the local community provide input on the aims of the research investigation, its methodology, and its anticipated outcome(s)?

The community mapping study done in the local community informs the next phase of the PhD project. The study was not a participatory research. When the 1^st^ author went to collect data in the local community the interview guide was already designed. Data were collected during COVID-19 which affected how the study methodology.

When engaging with the local community, how did you ensure that the informed consent documents and other materials could be understood by local stakeholders?

The author who collected data is local Motswana and informed consent forms were translated from English to the local language Setswana. Informed consent forms were also read and discussed with participants before they signed by local stakeholders.

Will the findings of the research be made available in an understandable format to stakeholders in the community where the study was conducted (e.g. via a presentation, summary report, copies of publications, etc.)? Please provide details of how this will be achieved.

Once the entire PhD project is done there will be a dissemination presentation in the community that will include the stakeholders, community members and community policy makers. Copies of the publication will also be shared to inform research, practice and policy.

**Non-human subjects research using specimens/ animals collected as part of the study, or those housed in archival collections. Examples include archaeology, paleontology, botany and zoology.**

Did the permission you obtained from a local authority to perform the study include an agreement on access to outputs and benefit sharing? This may include procedures to enable fair distribution of the benefits and resources arising from the research performed. Please include any details of Prior Informed Consent and Benefit Sharing Agreements obtained. These may be required by field-specific regulations, for example the Convention on Biological Diversity (CBD) and the associated Nagoya Protocol.

The authors did not use non-human subjects in the study.

If the material used in your study was imported, please A) provide the year it was imported and B) indicate whether permits were obtained to import/export the materials used, C) provide details of any permits obtained. If this information is not available, please indicate this.

The authors did not import any materials for the study.

If you used archival specimens, please state how the material used in your study was acquired by the institute it is held in and provide details of any permits obtained for the original excavations/ sample collection. If this information is not available, please indicate this.

The authors did not use archival specimens for the study.

How was the potential cultural significance of the materials collected in your study to local communities considered in your research design? Were Indigenous peoples and/or local researchers and institutions involved with archaeological excavations / collection of specimens? If so, please provide a description of their involvement.

The study did not collect any specimens from the community.

If your manuscript includes photographs of human remains please indicate whether authors obtained permission from descendants or affiliated cultural communities to do so.

The manuscript did not have any photographs.
