# Supplementary material for: Alcohol misuse within different socio-ecologies in rural communities of Botswana
Source: PLoS One. 2024 Sep 13;19(9):e0306542. doi: 10.1371/journal.pone.0306542 (PMC11398658; doi:10.1371/journal.pone.0306542)
Supplement: S1 File — Interview Guide For Participating In The Study. (DOCX) [file pone.0306542.s002.docx]

**ANNEX 5 (INTERVIEW GUIDE FOR STAKEHOLDERS)**

**INTERVIEW GUIDE FOR PARTICIPATING IN THE STUDY**

Thank you very much for agreeing to participate in this study as an adult whose childhood was influenced by an alcoholic. This interview will take about 40 to 60 minutes of your time

**NB:** The questions will be asked appropriately based on the right stakeholder in that office, such as the village chief, social worker, school guidance teacher

**Section A: Community leadership**

1. What is the population of this village?
2. a.) Who are the stakeholders that are present in this village? b) How do community members perceive the role of the said stakeholders?
3. What mode of transport is available for community members to access community services like social work offices, clinics, and schools?
4. What is the extent of influence the neighbouring villages have on this village population?
5. How do people access alcohol in the community?
6. a) How is alcohol sold in the community?
7. Is there unconventional selling of alcohol? If yes, how many places are there?
8. Are any of these sales problematic? Why?
9. Name some kind of alcohol that is available in the community.
10. Are people making a living by selling alcohol? How does it affect the different generations in that family?
11. How has been the situation of alcohol use since the measures to deal with COVID-19 were put in place?

**Section B: interview guide for Service providers**

1. What are the forms of alternative entertainment for youth and young adults in the village?
2. Could you tell me about alcohol-related challenges in the community, such as gender-based violence, school dropouts, teenage pregnancy, and rape cases?
3. Could you tell me about attitudes toward alcohol use in the community? How is alcohol problematic in the community?
4. Which population (age group, sex, occupation) is the most affected by alcohol use in the community?
5. How are they affected by alcohol use?
6. What types of alcohol are used in the community? Can you tell me about the pattern of alcohol use in the community?
7. What are the challenges faced by families with alcohol misuse among parents or caregivers?
8. What are the resources available in the village for people who are using alcohol to help them reduce the use of alcohol? Dikwaere, football, student clubs
9. How has been the situation of alcohol use since the measures to deal with COVID-19 were put in place?
10. What is the contribution of a service provider to alcohol use education in the village?

# **Additional questions for Kgosi (chief) and Village Development Committee members**

1. What is the history of bojalwa ja Setswana traditional / alcohol brew, and how is it made?
2. How is bojalwa ja Setswana traditional / alcohol brew used in cultural practices?
3. How is bojalwa ja Setswana traditional / alcohol brew used in families?
4. How is bojalwa ja Setswana traditional / alcohol brew used in cultural practices?
5. How is alcohol and beverages shared between generations?
6. Are there any laws that kgotla implements regarding alcohol use during the COVID-19 lockdown and alcohol burn period?
7. Was Kgotla involved in closing bars and shebeens during the COVID-19 lockdown and alcohol burn periods? Kindly share scenarios.
8. Does kgotla do anything about alcohol in the village? a) regarding illegal sales, b) regarding illegal alcohol use in the village, and what are the charges given?
9. What are interventions done so far by kgotla regarding alcohol use in the village
10. Are people making a living by selling alcohol? How does it affect the different generations in that family?
11. How has alcohol been understood in families? Are there any reported cases that can be shared as examples?
12. Any cases where there were generational alcohol use/ modern or traditional where caregivers shared with children?
13. What does the law say in such cases where caregivers are found giving a child alcohol?
14. What are the risks of alcohol use in families?
15. What are the challenges faced by families with alcohol use among parents or caregivers? Any reported cases of parents neglecting children due to alcohol use?
16. What resources exist for alcohol users in the community?
17. What is the history of bojalwa ja Setswana, how is it made, and what does it signify?
18. How is bojalwa ja Setswana used in families?
19. How is it used in cultural practices?
20. How is alcohol shared between generations?

# **Additional questions for Police**

1. What laws govern the traditional brews in communities?
2. What are the current legal frameworks that exist in Botswana regarding alcohol?
3. What is the current percentage of the alcohol levy, and how has it changed since 2011, when it was increased?
4. How are communities affected by alcohol in the village? Kindly share scenarios. How do children relate with adults on alcohol issues?
5. Do we have children loitering around bars, and what does the law say about this situation?
6. COVID-19 and alcohol sales: What are alcohol sales like during the COVID-19 lockdown?
7. What laws governed alcohol sales during lockdown? And what are the current laws that govern alcohol burn sales due to COVID-19?
8. Were there people who did not obey COVID-19 laws during lockdown and continued to drink alcohol? Kindly share scenarios of such incidents. What was the law regulations and the charges they got?
9. Any reported cases of alcohol sales during the COVID-19 lockdown and alcohol sale burn?
10. Are people making a living by selling alcohol in homes? How does it affect the different generations in that family?
11. How has alcohol been understood in families? Are there any reported cases that can be shared as examples?
12. Any cases where there were generational alcohol use/ modern or traditional where caregivers shared with children?
13. What does the law say in such cases where parents/caregivers are found giving a child alcohol?
14. What are the risks of alcohol use in families?
15. What are the challenges faced by families with alcohol use among parents or caregivers? Are there any reported cases of parents/ caregivers neglecting children due to alcohol use?
16. What resources exist for alcohol users in the community?

# **Additional questions to traditional alcohol sellers**

1. When were you born?

3. What are your working hours, and who are your regular customers?

4. Which types of traditional brews do you sell, and how do you prepare them? What have you brewed today?

5. How long have you been doing this type of business?

6. What can you say about the behaviour of customers?

8. How much do you measure the traditional alcohol you sell customers? How much money do you make daily in normal situations compared to now during COVID-19?

9. How has COVID-19 affected your alcohol-selling business?
